# Cognitive Performance as a Function of MAPT Haplotype: A Prospective Longitudinal Study of an Essential Tremor Cohort

**DOI:** 10.5334/tohm.768

**Published:** 2023-05-19

**Authors:** Ali Ghanem, Diane S. Berry, Kurt Farrell, Stephanie Cosentino, John F. Crary, Elan D. Louis

**Affiliations:** 1 Department of Neurology, University of Texas Southwestern Medical Center, Dallas, Texas, USA; 2 Department of Pathology, Department of Artificial Intelligence & Human Health, Nash Family Department of Neuroscience, Ronald M. Loeb Center for Alzheimer’s Disease, Friedman Brain Institute, Neuropathology Brain Bank & Research CoRE, Icahn School of Medicine at Mount Sinai, New York, USA; 3 Taub Institute for Research on Alzheimer’s Disease and the Aging Brain, New York, USA; 4 Department of Neurology, Columbia University Irving Medical Center, New York, USA

**Keywords:** essential tremor, genetic risk factors, MAPT H1, cognition, dementia

## Abstract

**Background:**

Cognitive impairment is a feature of essential tremor (ET). There are no studies of the genetic drivers of this association. We examined whether the microtubule-associated protein tau (MAPT) H1 haplotype is associated with cognitive performance in ET.

**Methods:**

ET cases genotyped for the MAPT H1 and H2 haplotypes completed a battery of neuropsychological tests at baseline and four follow-up evaluations. Chi-square, t-tests, and analyses of covariance examined associations between the presence of the MAPT H1 haplotype, cognitive diagnoses of normal, mild cognitive impairment (MCI), and dementia, and performance in specific cognitive domains.

**Results:**

We observed no evidence of cognitive differences as a function of the presence of the MAPT H1 haplotype. Specifically, cases with (n = 57) and without (n = 42) this haplotype did not differ with respect to the prevalence of diagnoses of MCI or dementia, *p* ≥ 0.87. Moreover, cases with vs without this haplotype did not differ in either the age or point in the disease course at which observed conversions to MCI or dementia occurred, *p*’s ≥ 0.51. Finally, no haplotype-related differences were observed in performance in the cognitive domains of attention, executive function, language, memory, visuospatial or global ability, *p*’s ≥ 0.21, or in changes in performance in these domains across time, *p*’s ≥ 0.08.

**Discussion:**

The study in an ET cohort revealed no influence of MAPT haplotypes on cognitive performance. This study serves as a valuable foundation for future studies to expand our understanding of the genetic drivers of cognitive impairment in ET.

**Highlights:**

This study found no evidence of cognitive differences between individuals with and without the MAPT H1 haplotype. Our work provides a valuable foundation for future work to expand our knowledge of the genetic drivers of cognitive impairment in ET.

## Introduction

Essential tremor (ET) is one of the most common movement disorders [1], affecting an estimated 2.2% of the entire US population [2] and resulting in considerable health care expenditures [3]. Kinetic tremor, typically in the hands and arms, is the prime clinical feature of this progressive disease [4,5]. In addition to tremor, ET is associated with cognitive decline [6]. Although ET cases display deficits in executive function and memory that worsen over time [6,7,8], and increased odds and risks of mild cognitive impairment (MCI) and dementia [9], the causes of such declines remain largely unexplored [6]. The identification of risk factors associated with cognitive decline in ET, if modifiable, could lead to target efforts at prevention and, even if not modifiable, would provide useful prognostic information to patients and their families. To date, no genetic risks have been identified, although exploration of these has been virtually nonexistent, leaving a gap in the literature.

Parkinson’s disease (PD) is a tremor disorder with links to ET [10]. Among these links are shared genetic predispositions in several studies [11,12,13]. Explorations of the risks underlying cognitive impairment in PD have implicated, among other entities, the microtubule-associated protein tau (MAPT) H1 haplotype [14]. The MAPT gene encodes tau, a cytoskeletal protein that contributes to neuronal polarity, building and stabilizing microtubules, and signal transduction [15]. In the context of neuropathology, distorted tau isoforms make up neurofibrillary tangles (NFTs), one of the features of primary tauopathies [15]. While ET is not considered a primary tauopathy, it is suggested that predisposition to tau pathology could play a role in the cognitive impairment observed in ET [16].

Two distinct haplotypes, H1 and H2, arise from an inversion in the chromosome 17q21 region, the location of the MAPT gene [15]. The H1 haplotype, known as MAPT H1, has been associated with poor cognitive performance [14] and conversion to dementia [17] in PD cohorts. To our knowledge, however, no studies have explored the association of MAPT haplotype variants to cognitive performance in an ET cohort.

Leveraging data from a prospective, longitudinal cohort study of ET cases, we assessed whether H1 homozygote ET cases (1) are more often diagnosed with MCI and dementia than are H2 haplotype carriers; (2) convert to MCI and dementia at a younger age or convert to MCI and dementia at an earlier point in the disease course than do H2 haplotype carriers; (3) perform more poorly on tests of attention, executive function, language, memory, visuospatial and global cognitive performance than do H2 haplotype carriers; and (4) show different patterns of performance across time in these domains than do H2 haplotype carriers. The overarching aim of these analyses was to attempt to shed further light on risks associated with cognitive decline in this highly prevalent neurological disease.

## Methods

### Overview

ET cases were enrolled and followed in a prospective, longitudinal study of cognitive performance (Clinical-Pathological Study of Cognitive Impairment in Essential Tremor; COGNET) from July 2014 through December 2021 [8,18]. Eligibility requirements include (1) a diagnosis of ET; (2) a baseline age of at least 55 years; and (3) no history of brain surgery as a treatment for ET. The study was approved by the Yale University, Columbia University, and University of Texas Southwestern Medical Center Institutional Review Boards. All cases provided written informed consent.

Cases took part in an initial baseline evaluation (Time 1), as well as a second (Time 2), third (Time 3), and fourth (Time 4) evaluation at 18, 36, and 54 months, respectively. A trained research assistant administered the evaluations during home visits. Each involved the completion of demographic/clinical questionnaires, a battery of neuropsychological tests, the collection of a blood or saliva sample, and a videotaped neurological examination. Based on the videotaped neurological examination, a senior movement disorders neurologist (E.D.L.) assigned clinical diagnoses of ET. These were derived from reliable [19] and valid [20] criteria and required at minimum, a moderate or greater amplitude kinetic tremor during three or more activities [21].

### Neuropsychological Test Battery

During each of the four study evaluations, cases completed a comprehensive battery of neuropsychological tests measuring cognitive performance in the domains of attention, executive function, language, memory, and visuospatial ability, as well as a measure of global cognitive performance. This battery included only assessments that required little or no reliance on motor functioning, minimizing any disadvantage to cases with moderate to severe tremor.

The following tests were administered for each domain: (1) *Attention*, measured via the Wechsler Adult Intelligence Scale IV (WAIS-IV) Digit Span Forward Test [22], and the Oral Symbol-Digit Modalities Test [23]; (2) *Executive function*, measured by the D-KEFS Sorting Test, the D-KEFS Verbal Fluency Test, the D-KEFS Color Word Interference Test, the D-KEFS Twenty Questions Test [24], and the Wechsler Adult Intelligence Scale IV (WAIS-IV) Digit Span Backward Test [22]; (3) *Language*, evaluated by scores on the Boston Naming Test [25] (4) *Memory*, assessed via the California Verbal Learning Test II [26], the Wechsler Memory Scale Revised: Logical Memory [27], and the Wechsler Memory Scale IV: Verbal Paired Associates Test [28]; and (5) *Visuospatial*, assessed by the Benton Judgement of Line Orientation [29], the Benton Facial Recognition Test [30], and the WAIS IV Visual Puzzles Test [22].

Finally, cases completed the Montreal Cognitive Assessment [31] (MOCA), which measures global cognitive performance.

### Cognitive Performance Measures

Following established protocols [32], aggregates of the aforementioned tests were constructed for each domain. Specifically, we converted the raw scores of each individual test to *z*-scores based on the means and standard deviations of cognitively normal participants (i.e., within sample normative scores). We then calculated the mean of the *z-*scores of tests used to assess a given domain, yielding an aggregate measure of performance for each.

MOCA scores provided an index of global cognitive performance.

### Cognitive Diagnosis Classifications: Normal, Mild Cognitive Impairment, Dementia

Cases were evaluated during a diagnostic consensus conference in which a neuropsychologist (S.C.) and a geriatric psychiatrist reviewed the comprehensive results of the evaluations of each case including neuropsychological test performance, and information gathered from the Clinical Dementia Rating (CDR) score [33]. For diagnostic purposes, raw scores associated with the previously described neuropsychological test battery were adjusted based on clinically normative data. On the basis of these data and following established protocols [18], cases were assigned a diagnosis of either normal, mild cognitive impairment (MCI), or dementia.

### Genotyping

DNA was isolated from either blood or saliva collected by a research assistant during a home visit. Samples were shipped to Mount Sinai Hospital and stored at room temperature (saliva) or in a –80°C freezer (blood). A 2 mL aliquot of each sample was packaged into a 5 mL Eppendorf tube, de-identified, and shipped to GENEWIZ (Azenta Life Sciences, South Plainfield, NJ), where DNA was robotically extracted, and the samples were genotyped using the Infinium Global Screening Array (Illumina, San Diego, CA). Raw idat files were converted to PLINK base files using genome studio (Illumina, San Diego, CA), and the rs1800547 single nucleotide polymorphism tag was analyzed to determine the MAPT haplotype. Specifically, the rs1800547 A allele is associated with the H1 haplotype, whereas the rs1800547 G allele defines the H2 haplotype. The inverted haplotype is rare outside of European Caucasian populations, limiting our study population to Caucasians; as the COGNET sample is 98% Caucasian, this requirement excluded very few cases from the analysis [34].

### Study Sample

Our sample consisted of 135 Caucasian cases who took part in all four evaluations. We excluded 36 cases for whom genotyping was not available, either because they refused to contribute a blood or saliva sample, or because sample collection was attempted, but unsuccessful. This yielded a final sample of 99 cases (60.6% female, mean age at baseline = 77.4 ± 8.6 years, mean age of tremor onset = 37.7 ± 21.4 years). All cases received a clinical diagnosis of ET at baseline. The mean time elapsed between baseline and follow-up (final) evaluation was 4.71 ± 0.32 years, range = 4.03 to 6.42 years.

For the purpose of the present analysis, we compared H1 homozygote cases (i.e., H1/H1 cases; defined as “H1/H1”) to H2 haplotype carriers (i.e., H1/H2 cases and H2/H2 cases; defined as “non-H1/H1”). Of our 99 cases, 57 (57.6%) were identified with an H1/H1 haplotype, and 42 (42.4%) with a non-H1/H1 haplotype. Of the latter group, 39 (39.4%) expressed an H1/H2 and 3 (3.0%) an H2/H2 haplotype.

### Statistical Analyses

We used Chi-square tests, independent sample t-tests, and Mann-Whitney tests to assess whether demographic or clinical characteristics distinguished ET cases expressing the H1/H1 haplotype from those expressing the non-H1/H1 haplotype (Table 1).

**Table 1:** Demographic and Clinical Characteristics as a Function of MAPT Haplotype.


	MAPT HAPLOTYPE^a^		

	H1/H1	NON-H1/H1	*p*

N	57	42	–

Baseline age (years)	77.9 ± 7.6	76.6 ± 9.9	0.43^b^

Sex (female)	32 (56.1)	28 (66.7)	0.29^c^

Race (Caucasian)	57 (100.0)	42 (100.0)	–

Education (years)	16.4 ± 2.4	15.1 ± 2.4	0.02 ^b^

Age of tremor onset (years)	39.1 ± 20.9	35.8 ± 22.0	0.46^d^

Tremor duration^e^ (years)	38.5 ± 21.7	40.5 ± 21.3	0.66^b^

Cognitive diagnosis^f^			

Normal cognition	45 (80.4)	32 (76.2)	0.87^c^

MCI	4 (7.1)	4 (9.5)	

Dementia	7 (12.5)	6 (14.3)	

Age at conversion to MCI (years)	83.1 ± 5.6	82.3 ± 11.5	0.84^b^

Age at conversion to dementia (years)	89.1 ± 7.8	88.5 ± 6.5	0.88^b^

Time elapsed from tremor onset to conversion to MCI (years)^g^	40.2 ± 24.3	46.8 ± 20.5	0.51^b^

Time elapsed from tremor onset to conversion to dementia (years)^h^	48.5 ± 26.0	51.2 ± 22.9	0.86^b^


*Note*: Sample N = 99. MCI = mild cognitive impairment. Degrees of freedom for specific measures may vary slightly due to missing data. Values indicate means ± standard deviation or N (percentages). ^a^ H1/H1 haplotype n = 57; non-H1/H1 haplotype n = 42. ^b^ Student’s t-test. ^c^ Chi square test. ^d^ Mann-Whitney test. ^e^ Baseline age minus tremor onset age. ^f^ Diagnostic classification at final evaluation. ^g^ Age at conversion to MCI minus age of tremor onset. ^h^ Age at conversion to dementia minus age of tremor onset.

Our first question was whether H1/H1 ET cases are more often diagnosed with MCI and dementia than are non-H1/H1 ET cases. To address this, chi-square statistics compared the proportions of H1/H1 cases assigned diagnoses of normal, MCI, and dementia at the fourth (final) evaluation with those observed for non-H1/H1 cases (Table 1).

Our second aim was to determine whether H1/H1 ET cases convert to MCI and dementia at a younger age or at an earlier point in the disease course than do non-H1/H1 ET cases. To this end, t-tests assessed whether either age of conversion to MCI, age of conversion to dementia, average time elapsed from tremor onset to conversion to MCI, or average time from tremor onset to conversion to dementia differed between H1/H1 and non-H1/H1 cases (Table 1).

Third, we examined whether H1/H1 ET cases perform more poorly on tests of attention, executive function, language, memory, visuospatial, or global cognitive performance than do non-H1/H1 ET cases. To this end, aggregate z-scores calculated for the five cognitive domains and MOCA scores were analyzed via separate 4 (time; evaluation 1, 2, 3, 4) × 2 (haplotype; H1/H1, non-H1/H1) analyses of covariance (ANCOVA), with repeated measures on the first factor, and baseline age and years of education as covariates (Table 2). The significance test associated with the main effect of haplotype directly addresses this question; a significant main effect would indicate that H1/H1 and non-H1/H1 cases differ in performance on a given measure, collapsing across evaluations (i.e., a between-groups comparison).

**Table 2:** Cognitive Performance as a Function of MAPT Haplotype and Time: Repeated Measures Analyses of Covariance.


DEPENDENT	MAPT	EVALUATION (TIME) MEAN				MAPT^b^		TIME^c^		INTERACTION^d^	
				
VARIABLE	HAPLOTYPE^a^	1	2	3	4	*F*	*p*	*F*	*p*	*F*	*p*

Attention	H1/H1	–0.17	–0.06	–0.22	–0.39	0.55	0.46	5.18	**0.002**	0.33	0.80

	Non-H1/H1	–0.26	–0.18	–0.25	–0.36						

	Total sample	–0.21	–0.11	–0.23	–0.38						

Executive Function	H1/H1	–0.12	–0.09	–0.17	–0.31	0.09	0.76	1.71	0.16	2.53	0.06

	Non-H1/H1	–0.25	–0.18	–0.18	–0.20						

	Total sample	–0.17	–0.13	–0.18	–0.26						

Language	H1/H1	–0.16	0.08	0.03	–0.15	2.41	0.12	2.27	0.08	0.13	0.94

	Non-H1/H1	–0.34	–0.12	–0.04	–0.33						

	Total sample	–0.24	–0.01	–0.01	–0.23						

Memory	H1/H1	–0.11	–0.14	0.10	–0.13	1.05	0.31	2.68	**0.05**	1.48	0.23

	Non-H1/H1	–0.22	–0.23	–0.14	–0.09						

	Total sample	–0.16	–0.18	0.01	–0.11						

Visuospatial	H1/H1	–0.10	0.04	–0.18	–0.28	0.38	0.54	4.41	**0.005**	0.83	0.48

	Non-H1/H1	–0.16	–0.01	–0.13	–0.16						

	Total sample	–0.12	0.02	–0.16	–0.23						

MOCA	H1/H1	25.0	24.6	23.4	22.6	0.05	0.83	12.46	**0.001**	0.21	0.89

	Non-H1/H1	24.9	24.7	23.9	23.0						

	Total sample	25.0	24.6	23.6	22.8						


*Note:* Sample N = 99. Individual analysis degrees of freedom vary slightly due to missing data. Analyses control for age at baseline and years of education. Bolded *p* values are significant at *p* ≤ 0.05. MOCA = Montreal Cognitive Assessment. ^a^ H1/H1 haplotype n = 57; Non-H1/H1 haplotype n = 42. ^b^ *F*-ratio, *p* for main effect of MAPT haplotype, collapsing across time (evaluation). ^c^ *F*-ratio, *p* for main effect of time (evaluation), collapsing across haplotype. ^d^ *F*-ratio, *p* for interaction of MAPT haplotype and time.

Our final aim was to assess whether H1/H1 and non-H1/H1 cases exhibit different patterns of performance across time on any of the five cognitive domains or MOCA scores. This issue is directly addressed by the significance test associated with the interaction of time and haplotype yielded by the previously described ANCOVA (Table 2); a significant interaction would indicate different patterns of performance on a given dependent variable across time for the H1/H1 and non-H1/H1 cases (i.e., a comparison of the longitudinal effects observed within each group).

## Results

### Demographic and Clinical Characteristics

Analyses revealed no significant differences between the baseline age, sex distribution, age of tremor onset, or tremor duration observed for H1/H1 and non-H1/H1 cases, all *p*’s ≥ 0.29. Cases in the H1/H1 group reported significantly more years of education than did those in the non-H1/H1 group, *M*’s = 16.4 and 15.1, respectively, *p* = 0.02 (Table 1).

### Prevalence of Normal, MCI, and Dementia Diagnoses

The distribution of cognitive diagnoses (normal, MCI, dementia) did not differ at the final (Time 4) evaluation as a function of the haplotype group, *X*^2^ = 0.28, *p* = 0.87 (Table 1). Thus, diagnoses of MCI or dementia were not more prevalent among H1/H1 cases than among non-H1/H1 cases.

### Age and Point of Disease Progression at Conversion to MCI and Dementia

Neither age at conversion to MCI nor age at conversion to dementia differed between H1/H1 and non-H1/H1 cases, *t*’s ≤ 0.21, *p*’s ≥ 0.84 (Table 1). H1/H1 cases and non-H1/H1 cases also did not differ in either time elapsed between age of tremor onset and age at conversion to MCI, *t* = –0.67, *p* = 0.51, or in time elapsed between age of tremor onset and age at conversion to dementia, *t* = –0.18, *p* = 0.86 (Table 1). In sum, H1/H1 cases who did convert to either MCI or dementia did not do so at a younger age or an earlier point in the disease course than non-H1/H1 cases.

### Cognitive Performance

Separate ANCOVAs performed on the measures of attention, executive function, language, memory, visuospatial performance, and MOCA scores revealed no significant main effects of haplotype for any measure of cognitive performance, all p’s ≥ 0.21 (Table 2). Thus, cases expressing the H1/H1 haplotype did not perform differently from non-H1/H1 cases on any assessment of cognitive performance.

### Cognitive Performance Across Time

The ANCOVAs further revealed no significant interactions of time and haplotype for any of the six cognitive performance variables, although the time by haplotype interaction only did approach significance for executive function, *F* (3, 285) = 2.53, *p ≥* 0.06 (Table 2). Thus, H1/H1 and non-H1/H1 ET cases did not exhibit significantly different patterns of cognitive performance across time on any measure (Table 2).

## Discussion

To our knowledge, this is the only study that examines whether cognitive performance in an ET cohort differs as a function of MAPT haplotypes. Specifically, diagnostically, analyses reveal that ET cases with and without H1/H1 haplotype did not differ in the distribution of diagnoses of normal, MCI and dementia, in the age at which cases converted to either MCI or dementia, or in the time elapsed between age of tremor onset and conversion to either MCI or dementia. Moreover, in terms of specific cognitive domains, H1/H1 and non-H1/H1 ET cases did not differ in attention, executive function, language, memory, visuospatial performance, or global cognition (MOCA) scores. Furthermore, the data provide no evidence of any MAPT haplotype-linked differences in patterns of change in performance across time in any of these six cognitive performance scores.

Although this is the only study of its kind carried out with a sample of ET cases, a large body of work examines the link between MAPT haplotypes and cognitive performance in PD. However, many of these studies have methodological limitations, and together yield mixed results [14,35,36]. For example, a study by Williams-Gray et al., a commonly cited paper on the topic, reports that PD cases with the H1/H1 haplotype exhibit greater cognitive decline and are at higher risk for developing dementia than those with the H2 haplotype [14]. Conversely, Paul et al. found no association between the MAPT H1 haplotype and changes in Mini-Mental State Examination [37] (MMSE) scores over time in a cohort of PD cases [35]. A limitation of both analyses is that cognitive performance was only assessed by the MMSE, which does not sample all cognitive domains [38]. Other studies that include a more comprehensive range of cognitive measures found no influence of the MAPT H1 haplotype on cognitive function among PD cohorts [35,36,39]. For example, a study of a large cohort of PD cases reported no significant associations between MAPT H1 haplotype and nine different psychometric tests of cognition [36]. Overall, the data on the effect of the MAPT H1 haplotype on cognitive performance in PD is not entirely conclusive.

Our study is not without limitations. First, a larger sample would be desirable. The present work was limited by a relatively small, fixed sample size of 99, and one could miss a small true effect with this number of cases. Therefore, replications with additional samples are highly desirable. Second, our cases were highly educated (a mean of 15+ years in both haplotype groups), a known contributor to performance across most cognitive measures [40]. Moreover, as cases in our sample completed four evaluations over a period of nearly five years, they may have been somewhat healthier and more socially engaged than a random sample of ET patients. We encourage researchers to recruit more diverse samples of ET cases in future studies, when possible, to evaluate the generalizability of the results.

Although this study has some limitations, it possesses a number of notable strengths. First, the ET diagnosis required for inclusion in the study was based on a detailed neurological examination by a senior movement disorders neurologist. Additionally, multiple detailed assessments evaluated by a neuropsychologist and geriatric psychiatrist were used to determine cognitive performance and cognitive diagnoses. Moreover, the cognitive assessments utilized in the study require little or no reliance on motor functioning, ruling out the interference of motor symptoms with cognitive test performance.

This is the first longitudinal exploration of the influence of MAPT haplotypes on cognitive performance in an ET cohort. No differences in cognitive performance or in diagnoses distinguished ET cases with the MAPT H1 haplotype from those without the variant, suggesting that the MAPT H1 haplotype does not influence the cognitive performance of individuals with ET. Although replication of this work is needed, it serves as a valuable foundation for future longitudinal studies to expand our understanding of the genetic drivers of cognitive impairment in ET.
